# Diagnostic Ability of Macular Ganglion Cell Inner Plexiform Layer Measurements in Glaucoma Using Swept Source and Spectral Domain Optical Coherence Tomography

**DOI:** 10.1371/journal.pone.0125957

**Published:** 2015-05-15

**Authors:** Zhiyong Yang, Andrew J. Tatham, Robert N. Weinreb, Felipe A. Medeiros, Ting Liu, Linda M. Zangwill

**Affiliations:** 1 Hamilton Glaucoma Center and Department of Ophthalmology, University of California San Diego, La Jolla, California, United States of America; 2 Princess Alexandra Eye Pavilion and Department of Ophthalmology, University of Edinburgh, Edinburgh, United Kingdom; 3 Department of Ophthalmology, Daping Hospital, Third Military Medical University, Chongqing, China; Universidade Federal do Rio de Janeiro, BRAZIL

## Abstract

**Purpose:**

To evaluate the diagnostic ability of macular ganglion cell and inner plexiform layer measurements in glaucoma, obtained using swept source (SS) and spectral domain (SD) optical coherence tomography (OCT) and to compare to circumpapillary retinal nerve fiber layer (cpRNFL) thickness measurements.

**Methods:**

The study included 106 glaucomatous eyes of 80 subjects and 41 eyes of 22 healthy subjects from the Diagnostic Innovations in Glaucoma Study. Macular ganglion cell and inner plexiform layer (mGCIPL), macular ganglion cell complex (mGCC) and cpRNFL thickness were assessed using SS-OCT and SD-OCT, and area under the receiver operating characteristic curves (AUCs) were calculated to determine ability to differentiate glaucomatous and healthy eyes and between early glaucomatous and healthy eyes.

**Results:**

Mean (± standard deviation) mGCIPL and mGCC thickness were thinner in both healthy and glaucomatous eyes using SS-OCT compared to using SD-OCT. Fixed and proportional biases were detected between SS-OCT and SD-OCT measures. Diagnostic accuracy (AUCs) for differentiating between healthy and glaucomatous eyes for average and sectoral mGCIPL was similar in SS-OCT (0.65 to 0.81) and SD-OCT (0.63 to 0.83). AUCs for average cpRNFL acquired using SS-OCT and SD-OCT tended to be higher (0.83 and 0.85, respectively) than for average mGCC (0.82 and 0.78, respectively), and mGCIPL (0.73 and 0.75, respectively) but these differences did not consistently reach statistical significance. Minimum SD-OCT mGCIPL and mGCC thickness (unavailable in SS-OCT) had the highest AUC (0.86) among macular measurements.

**Conclusion:**

Assessment of mGCIPL thickness using SS-OCT or SD-OCT is useful for detecting glaucomatous damage, but measurements are not interchangeable for patient management decisions. Diagnostic accuracies of mGCIPL and mGCC from both SS-OCT and SD-OCT were similar to that of cpRNFL for glaucoma detection.

## Introduction

The diagnosis of glaucoma in clinical practice largely depends on identification of characteristic structural changes to the optic nerve head, which are often accompanied by functional deficits on visual field testing.[1,2] As glaucomatous visual loss is irreversible, early diagnosis is important; however, this can be challenging due to large inter-individual variation in normal disc appearance, inter-observer differences in disc evaluation, and lack of sensitivity of visual field testing due to physiological redundancy in retinal ganglion cell receptive fields.[3,4]

Recent advances in imaging technologies, especially the development of optical coherence tomography (OCT), provide a means for the objective evaluation of structural changes to the optic nerve head and retina in glaucoma, and offer the potential for improved detection of disease. It has been shown that circumpapillary retinal nerve fiber layer (cpRNFL) measurements from OCT have good ability to differentiate glaucomatous and healthy eyes with AUC ranging from 0.728 to 0.969.[5–9] [10]

Although cpRNFL thinning is a useful marker of glaucomatous damage, there is growing recognition that measurement of the glaucomatous macula may also reveal changes that could potentially aid diagnosis.[11] The macula is essential for good central vision and therefore glaucomatous changes in this region may have particularly serious consequences for vision related quality of life and daily function. Furthermore, as approximately 50% of all retinal ganglion cells are sited within 10 degrees of the fovea, presuming an absence of macular co-morbidities, changes in macular structure are likely to be a good indication of glaucoma-related neural losses. [12] Measurement of macular retinal ganglion cell-related structure may therefore offer a plausible adjunct or alternative to traditional circumpapillary measurements for glaucoma diagnosis. As the macula is largely devoid of large vessels and has a readily identifiable center, assessment of the macula may also overcome some limitations of circumpapillary measurements, such as interference from retinal and optic nerve head vasculature, parapapillary atrophy, and variable placement of the measurement circle around the disc.

Recent studies have shown that OCT measurements of macular structures such as macular ganglion cell complex (mGCC) may be useful for differentiating healthy and glaucomatous eyes.[13–17] mGCC has been reported to have similar diagnostic performance to cpRNFL,[13–17] however, some studies have suggested measurement of cpRNFL to be better.[18,19] The mGCC thickness measurement incorporates several retinal layers, including ganglion cell layer, inner plexiform layer and overlying retinal nerve fiber layer. It is possible that finer segmentation of the ganglion cell-containing retinal layers alone might facilitate better detection of glaucomatous damage, particularly as it is loss of retinal ganglion cells that is the defining histological feature of glaucoma. Recent developments in SD-OCT provide the ability for segmentation of the ganglion cell containing macular ganglion cell inner plexiform layer (mGCIPL).

Conventionally, it is necessary to perform two separate scans, one for macular, and one for circumpapillary measurements. A new OCT technology, swept-source OCT (SS-OCT), which has a faster scan speed and longer wavelength than SD-OCT, allows a wide-angle scan protocol to image a larger 12 X 9 mm area of the posterior pole thereby including optic nerve head and macula in a single scan, with the capability to segment both mGCIPL and cpRNFL.

The primary aim of the current study was to evaluate the ability of mGCIPL and mGCC measurements to differentiate glaucomatous from healthy eyes. mGCIPL and mGCC measurements were obtained using both SD-OCT and SS-OCT with the aim of comparing the diagnostic ability of the two devices. As the diagnostic ability of different layers may differ depending on the stage of disease, we also evaluated the performance of these devices in eyes with early glaucoma and compared performance to cpRNFL measurements by both OCT devices.

## Methods

### Ethics Statement

This was a cross-sectional observational study of participants from the Diagnostic Innovations in Glaucoma Study (DIGS) (clinicaltrial.gov identifier: NCT00221897, National Eye Institute) at the University of California San Diego (UCSD). DIGS is a prospective longitudinal study designed to evaluate optic nerve structure and visual function in glaucoma. Written informed consent was obtained from all participants, and UCSD Human Research Protection Program Institutional Review Board prospectively approved all methods (IRB # 140276). All study methods adhered to the tenets of the Declaration of Helsinki for research involving human subjects and the study was conducted in accordance with the regulations of the Health Insurance Portability and Accountability Act.

### Ocular examination and glaucoma definition

Methodological details of the DIGS have been described in detail previously.[20] In brief, at each semi-annual visit during follow-up, subjects underwent a comprehensive ophthalmologic examination including review of medical history, blood pressure, best-corrected visual acuity, slit-lamp biomicroscopy, intraocular pressure measurement, gonioscopy, and standard automated perimetry (SAP) using the Swedish Interactive Threshold Algorithm (Standard 24–2). The study included only subjects with open angles on gonioscopy. Subjects were excluded if they presented with a best-corrected visual acuity less than 20/40, spherical refraction outside ± 5.0 diopters or cylinder correction outside 3.0 diopters, or any other ocular or systemic disease that could affect the optic nerve or visual field.

Eyes were classified as glaucomatous if they had repeatable (≥3 consecutive) abnormal SAP test results and/or documented evidence of progressive disc damage on masked grading of stereophotographs, with or without an abnormal SAP result. Healthy subjects were recruited from the general population through advertisements or from the staff and employees at UCSD. Healthy subjects had IOP less than 22 mmHg with no history of increased IOP, normal SAP and disc appearance in both eyes.

### Standard automated perimetry

SAP was performed using the Humphrey Field Analyzer II (Carl Zeiss Meditec, Dublin, CA, USA) and the 24–2 Swedish interactive threshold algorithm (SITA Standard 24–2, Carl Zeiss Meditec, Inc., Dublin, CA, USA). All visual fields were evaluated by the UCSD Visual Field Assessment Center (VisFACT).[21] Visual fields with more than 33% fixation losses or false-negative errors, or more than 15% false-positive errors, were excluded. The only exception was the inclusion of visual fields with false-negative errors of more than 33% when the field showed advanced disease. SAP tests were defined as normal if the mean deviation (MD) and pattern standard deviation (PSD) were within 95% normal confidence limits and the Glaucoma Hemifield Test (GHT) was also within normal limits. An abnormal SAP test was defined as a visual field with a PSD with P <0.05 and/or a GHT outside normal limits. Early glaucoma was defined as a MD of ≥ -6 dB.

### Optic disc stereophotographs

Simultaneous stereoscopic optic disc photography was performed for all subjects at their annual dilated examination. Digitized film and digital stereoscopic images (Kowa Nonmyd WX3D, software version VK27E, Kowa Company Ltd, Tokyo Japan) were reviewed with a stereoscopic viewer (Screen-VU stereoscope, PS Mfg., Portland, Oregon, USA) by two or more experienced graders. Each grader was masked to the subject's identity and to the other test results. Details of the methodology employed to grade optic disc photographs as glaucomatous at the UCSD Optic Disc Reading Center have been provided previously.[20,22] Glaucomatous appearance of the optic disc was defined as evidence of excavation, neuroretinal rim thinning or notching, localized or diffuse retinal nerve fiber layer defect, or a between-eye asymmetry of the vertical cup-disc ratio more than 0.2.

### Optical Coherence Tomography

**Fig 1:**
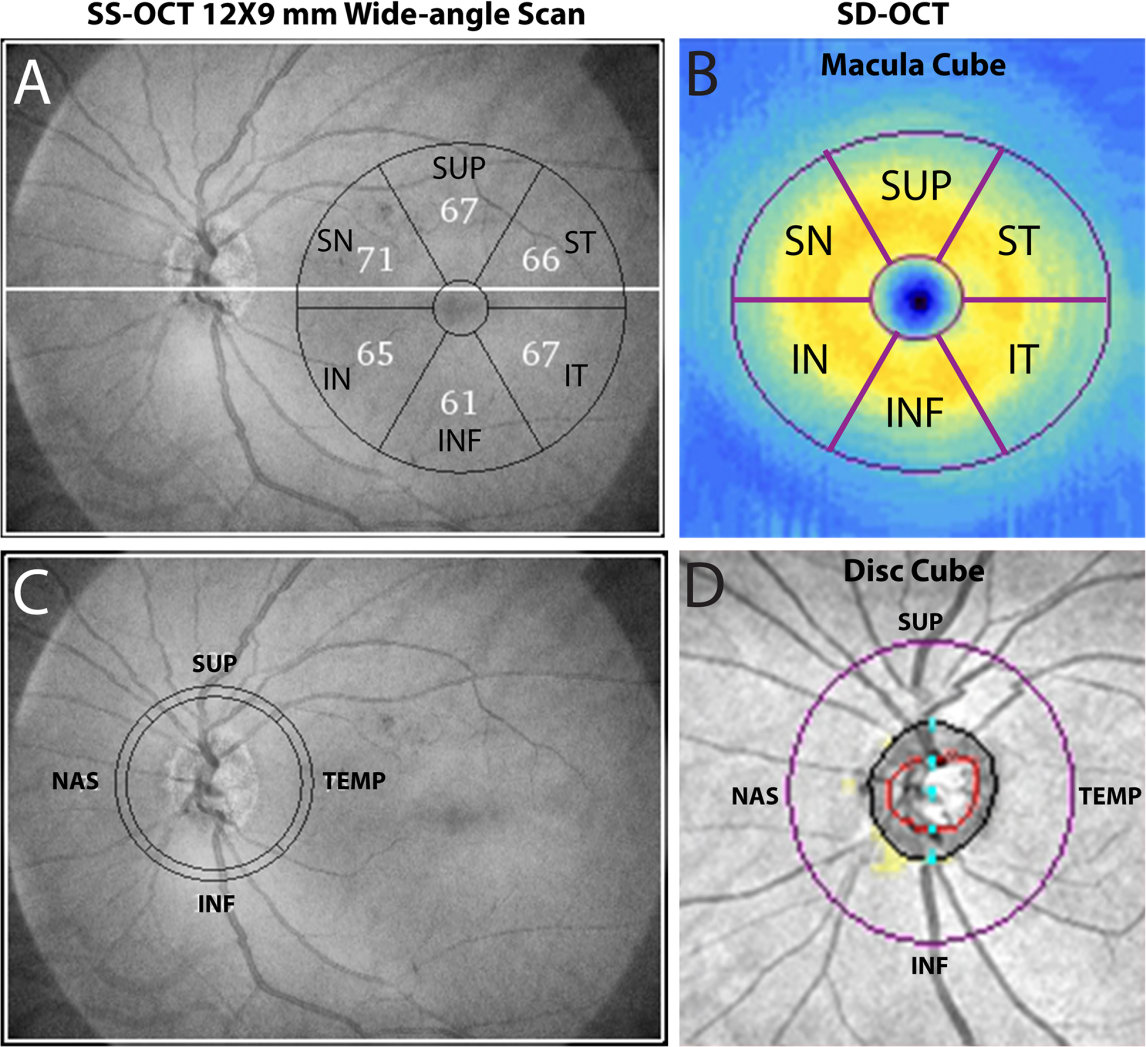
Measurement of macular ganglion cell inner plexiform layer (mGCIPL), ganglion cell complex (mGCC), circumpapillary retinal nerve fiber layer (cpRNFL) by swept source optic coherence tomography (SS-OCT) wide-angle scan and spectral domain optic coherence tomography (SD-OCT) macular and disc cube scans.

mGCIPL, mGCC, and cpRNFL thicknesses were assessed from images acquired using SS-OCT (Deep Range Imaging OCT, Topcon, Tokyo, Japan), and SD-OCT (Cirrus HD-OCT, Model 5000, software version 4.5–6.0, Carl Zeiss Meditec) on the same day or within 5 weeks. The Deep Range Imaging OCT is a SS-OCT device that uses a wavelength-sweeping laser with a center wavelength of 1050 nm and a tuning range of approximately 100 nm.[23] For the present study, all eyes were imaged using the wide-angle (12 x 9 mm) scan setting centered on the posterior pole (Fig 1). It was therefore possible to obtain images of the macular and optic nerve head in a single scan. The 12 x 9 mm scan comprises 256 B-scans, each comprising 512 A-scans for a total of 131,072 axial scans/volume. 100,000 A-scans are acquired per second for optical axial resolution of 8 μm and lateral resolutions 20 μm, for the wide-angle scan.[24] The total acquisition time was 1.3 seconds per 12 x 9 mm scan.

SS-OCT segmentation software (version 9.12) was used to identify the limits of the RNFL and IPL, and to determine the thickness of mGCIPL, mGCC, and cpRNFL, throughout the scan. mGCIPL thickness includes the ganglion cell layer and the inner plexiform layer IPL; mGCC thickness includes the GCIPL and the RNFL. Data was exported using the manufacturer’s OCT-Batch utility (version 4.3.0.118). The quality of each scan and accuracy of the segmentation algorithm was independently reviewed by masked reviewers (Z.Y. and T.L.). Eight eyes were excluded due to clipped, poorly focused images, and images with segmentation failure and motion artifacts. To measure mGCIPL and mGCC thickness, a circle of 6 mm in diameter was automatically centered on the fovea (Fig 1A). Average and sectoral mGCIPL and mGCC thicknesses were measured after segmentation with the same software (version 9.12). Foveal placement was manually adjusted when automated placement was not accurate. For cpRNFL measurement, a circumpapillary circle of 3.4 millimeter in diameter was automatically placed, and centered on optic disc, and RNFL thickness along the circle was determined after segmentation. (Fig 1C).

Macular and cpRNFL measures were also acquired separately two cube scans, the optic disc cube scan and macula cube scan with Cirrus HD-OCT (Fig 1B and 1D), which uses a superluminescent diode laser with a center wavelength of 840 nm and an acquisition rate of 27,000 A-scans per second. The axial and lateral resolutions are 5 μm and 30 μm, respectively. The protocol used for evaluating mGCIPL and mGCC thickness was macula cube, which obtained a 3-dimensional scan of a 6 X 6 mm area centered on the fovea. Ganglion cell analysis (GCA) algorithm in Cirrus analysis/export software version 6.5 was used to segment a 6 X 6 X 2 mm area defined by an elliptical annulus centered on the fovea. The GCA algorithm is able to segment and measure mGCIPL and mGCC. To obtain cpRNFL measurement, the optic disc cube protocol was used to scan a 6 X 6 mm area centered on the disc. Data from a tridimensional cube of 200 X 200 X1024 (depth) points was collected, and cpRNFL measurements along a 3.4 mm circle automatically provided. All Cirrus images were reviewed by the UCSD Imaging Data Evaluation and Analysis (IDEA) Center to ensure the scan was centered, that the signal strength was > 6 dB and that there were no artifacts. Approximately 14% of scans were excluded because they were inverted, clipped, showed signs of eye movement or had coexistent retinal pathological abnormalities. Foveal placement was manually adjusted when the automated location was inaccurate.

### Statistical Analysis

Normality assumption was assessed by inspection of histograms and using Shapiro-Wilk tests. Student *t*-tests and Chi square tests were used for group comparison for normally distributed variables. Receiver operating characteristic (ROC) curves were constructed to assess the ability of each parameter to distinguish subjects with glaucoma from healthy controls. A ROC curve is a plot of the true-positive rate (TPR) versus the false-positive rate (FPR) for all possible cut-points. The area under the ROC curve (AUC) was used to summarize the diagnostic accuracy of each parameter. An AUC of 1.0 represents perfect discrimination, whereas an area of 0.5 represents chance discrimination.[25,26] ROC curves were adjusted for age differences between cases and controls using a previously described ROC regression technique.[27,28] Confidence intervals were obtained using a bootstrap resampling procedure (*n* = 1000 resamples). Observations from two eyes of the same subject are likely to be correlated, which can lead to underestimation of true variance. A between-cluster variance estimator was therefore used to account for correlations between eyes of the same subject and calculate robust variance estimates. [29] All statistical analyses were performed with commercially available software (Stata version 13; StataCorp, College Station, TX).

Bland-Altman plots were used to determine the agreement between corresponding average and sectoral measures of SS-OCT and SD-OCT.[30] The differences for each pairwise comparison were plotted against their mean. A fixed bias was present when there was a systemic difference in the corresponding measurements of the two devices. Mean differences and their 95% confidential intervals were calculated. A proportional bias was present if the difference of pairwise comparison correlated with their mean. To evaluate the correlation between the two devices, we calculated coefficients of determination (R^2^) for each pairwise comparison.

Dataset for this study was presented in S1 Table.

## Results

A cohort of 147 eyes of 102 participants was included in this study, among them 106 eyes were diagnosed of having glaucoma and 41 were healthy eyes. Among 106 eyes of glaucoma patients, 66 were diagnosed by the development of at least three consecutive abnormal SAPs alone, 11 by progressive glaucomatous change on disc stereophotograph alone, and 29 eyes were positive for both criteria. Table 1 summarizes the demographic and clinical characteristics of the participants. Patients with glaucoma were significantly older than healthy subjects (70.6 ± 12.6 vs. 50.4 ± 12.0 years, P < 0.001) but there was no difference in gender or ancestry between groups. Glaucomatous eyes had an average SAP MD of -5.23 ± 5.60 dB and PSD of 5.96 ± 3.85 dB.

**Table 1:** Demographics and ocular data of healthy participants and glaucoma patients Results for mean ± standard deviation.

		Healthy	Glaucoma		Early glaucoma	
**By patient**		**n = 22**	**n = 80**	***P* value**	**n = 56**	***P* value** *
Age (years)^a^		50.4 ± 12.0	70.6 ± 12.6	< 0.001	69.4 ± 13.2	< 0.001
Gender (%)^b^		68.2%	53.8%	0.226	60.7%	0.539
Ancestry (%)^b^	Caucasian	40.9%	58.8%	0.401	57.1%	0.397
	African	36.3%	30.0%	0.401	30.4%	0.397
	Other	22.8%	11.2%	0.401	12.5%	0.397
**By eye**		**n = 41**	**n = 106**	***P* value**	**n = 68**	***P* value** *
IOP (mmHg) ^a^		14.2 ± 1.9	14.1 ± 4.0	0.839	15.4 ± 3.3	0.019
MD (dB)^a^		0.39 ± 1.02	-5.70 ± 5.69	< 0.001	-2.4 ± 1.7	< 0.001
PSD (dB)^a^		1.45 ± 0.32	5.96 ± 3.85	< 0.001	3.6 ± 2.2	< 0.001

^a^ Two-sample t test,

^b^ Chi square test,

* Healthy vs. Early glaucoma

Abbreviations: IOP = intraocular pressure; MD = mean deviation; PSD = pattern standard deviation; dB = decibels.

The macular and circumpapillary measurements from SS-OCT and SD-OCT, including mGCIPL, mGCC and cpRNFL thickness, are shown in Table 2. Average mGCIPL thickness was 70.5 ± 5.5 μm versus 60.4 ± 7.2 μm in healthy and glaucomatous eyes respectively by SS-OCT (P < 0.001), and 82.1 ± 6.6 μm versus 67.9 ± 10.0 μm respectively using SD-OCT (P < 0.001). In healthy subjects, the thickest sectoral mGCIPL measurement was superonasal, whereas the inferior sector had thinnest mGCIPL measurement by both devices. Global and all sectoral mGCIPL thicknesses were significantly reduced in those with glaucoma compared to healthy eyes (P < 0.001 for all sectors, Table 2).

**Table 2:** Macular ganglion cell inner plexiform layer thickness, macular ganglion cell complex thickness and circumpapillary retinal nerve fiber layer thickness measurements by swept source optical coherence tomography and spectral domain optic coherence tomography.

	SS-OCT					SD-OCT				
**mGCIPL (**μm)mean ± SD	**Control n = 41**	**Glaucoma n = 106**	**P value**	**Early Glaucoma n = 68**	**P value** *	**Control n = 41**	**Glaucoma n = 106**	**P value**	**Early Glaucoma n = 68**	**P value** *
AVG	70.5 ± 5.5	60.4 ± 7.2	< 0.001	62.3 ± 6.5	< 0.001	82.1 ± 6.6	67.9 ± 10.0	< 0.001	71.3 ± 8.9	< 0.001
SUP	68.7 ± 6.1	58.8 ± 8.2	< 0.001	60.2 ± 7.5	< 0.001	82.8 ± 7.3	69.4 ± 11.6	< 0.001	72.4 ± 10.6	< 0.001
SN	73.2 ± 6.0	62.6 ± 9.9	< 0.001	65.1 ± 7.9	< 0.001	84.1 ± 7.1	72.0 ± 11.8	< 0.001	74.9 ± 9.7	< 0.001
ST	71.7 ± 5.5	63.7 ± 6.6	< 0.001	65.1 ± 6.3	< 0.001	80.6 ± 6.2	67.5 ± 10.5	< 0.001	70.3 ± 9.7	< 0.001
INF	65.8 ± 6.1	55.1 ± 7.9	< 0.001	56.9 ± 7.5	< 0.001	80.7 ± 7.5	64.7 ± 11.4	< 0.001	68.6 ± 10.6	< 0.001
IN	71.1 ± 6.0	59.8 ± 9.3	< 0.001	62.8 ± 7.9	< 0.001	82.0 ± 6.9	68.9 ± 12.2	< 0.001	72.8 ± 10.5	< 0.001
IT	72.7 ± 5.3	62.3 ± 6.8	< 0.001	63.8 ± 6.6	< 0.001	82.3 ± 6.6	64.8 ± 10.5	< 0.001	68.4 ± 9.7	< 0.001
Minimum	N/A	N/A	N/A	N/A	N/A	79.5 ± 6.3	58.9 ± 11.4	< 0.001	64.2 ± 11.3	< 0.001
**mGCC (**μm)mean ± SD	**Control**	**Glaucoma**	**P value**	**EG**	**P value** *	**Control**	**Glaucoma**	**P value**	**EG**	**P value** *
AVG	107.9 ± 8.1	88.3 ± 12.8	< 0.001	93.1 ± 10.7	< 0.001	114.6 ± 7.2	94.8 ± 14.7	< 0.001	100.2 ± 12.4	< 0.001
SUP	108.4 ± 9.0	90.1 ± 15.1	< 0.001	94.6 ± 12.3	< 0.001	118.4 ± 8.4	99.7 ± 17.6	< 0.001	104.6 ± 14.6	< 0.001
SN	118.3 ± 8.0	101.8 ± 16.5	< 0.001	105.9 ± 13.8	< 0.001	121.5 ± 7.7	105.2 ± 17.7	< 0.001	109.4 ± 14.0	< 0.001
ST	96.6 ± 6.9	80.3 ± 12.6	< 0.001	84.2 ± 10.3	< 0.001	104.0 ± 6.3	87.2 ± 14.3	< 0.001	91.5 ± 12.1	< 0.001
INF	106.9 ± 10.0	83.7 ± 14.2	< 0.001	89.3 ± 12.5	< 0.001	117.1 ± 8.4	92.1 ± 18.3	< 0.001	99.2 ± 16.3	< 0.001
IN	117.8 ± 10.2	96.0 ± 16.4	< 0.001	102.1 ± 14.2	< 0.001	120.7 ± 7.8	100.5 ± 17.9	< 0.001	107.2 ± 14.7	< 0.001
IT	99.4 ± 7.8	77.8 ± 12.7	< 0.001	82.5 ± 11.4	< 0.001	106.7 ± 6.5	83.7 ± 15.2	< 0.001	89.1 ± 13.8	< 0.001
MIN	N/A	N/A	N/A	N/A	N/A	94.5 ± 7.0	70.2 ± 14.6	< 0.001	75.5 ± 14.2	< 0.001
**cpRNFL (**μm)mean ± SD	**Control**	**Glaucoma**	**P value**	**EG**	**P value** *	**Control**	**Glaucoma**	**P value**	**EG**	**P value** *
Average	102.4 ± 15.6	71.1 ± 18.2	< 0.001	79.3 ± 14.3	< 0.001	95.3 ± 8.6	72.4 ± 12.1	< 0.001	76.5 ± 11.0	< 0.001
SUP	125.6 ± 20.4	84.4 ± 23.9	< 0.001	93.0 ± 20.8	< 0.001	121.8 ± 13.5	86.9 ± 18.1	< 0.001	92.3 ± 16.2	< 0.001
INF	131.6 ± 23.5	82.5 ± 29.0	< 0.001	95.5 ± 25.0	< 0.001	125.5 ± 16.5	84.5 ± 22.3	< 0.001	92.6 ± 21.2	< 0.001
NAS	75.2 ± 15.2	58.3 ± 20.4	< 0.001	63.9 ± 15.3	< 0.001	69.2 ± 8.1	66.0 ± 10.4	< 0.001	66.6 ± 11.2	< 0.001
TEMP	77.3 ± 18.0	59.1 ± 16.2	< 0.001	64.6 ± 12.7	< 0.001	64.9 ± 12.5	52.4 ± 11.2	< 0.001	54.8 ± 10.1	< 0.001

Abbreviations: SS-OCT = swept source optic coherence tomography; SD-OCT = spectral domain optic coherence tomography; mGCIPL = macular ganglion cell inner plexiform layer; mGCC = macular ganglion cell complex; cpRNFL = circumpapillary retinal nerve fiber layer; EG = early glaucoma; SD = standard deviation; AVG = average; SUP = superior; INF = inferior; NAS = nasal; TEMP = temporal; SN = superonasal; IN = inferonasal; ST = superotemporal; IT = inferotemporal; MIN = minimum.

* Healthy vs. EG.

**Fig 2:**
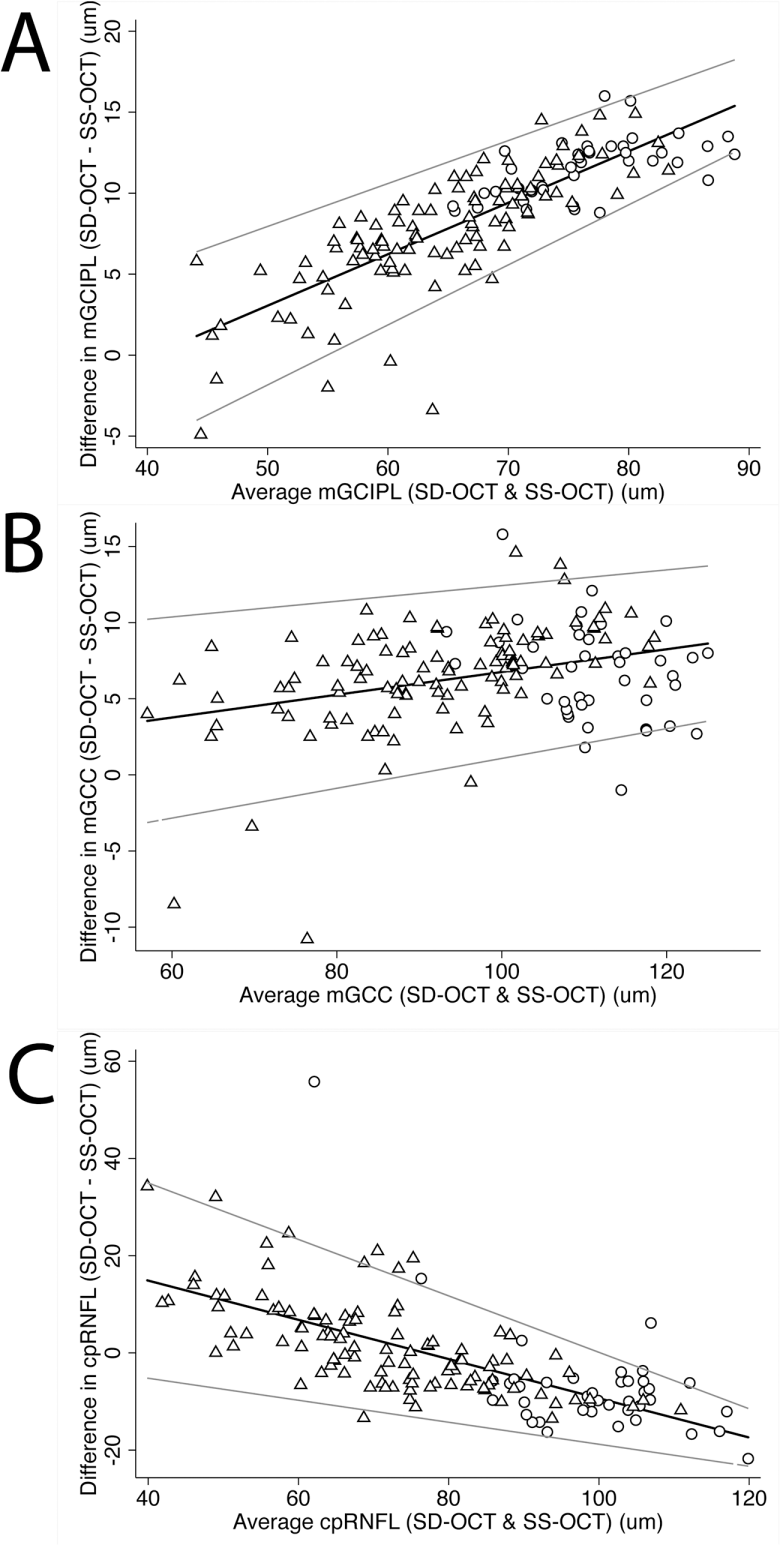
Bland-Altman plots showing the agreement between SS-OCT and SD-OCT. A: mGCIPL; B: mGCC; C: cpRNFL. Circle: healthy eyes; Triangle: eyes of glaucoma patients.

There was good correlation between mGCIPL thickness using SS-OCT and SD-OCT (R^2^ = 0.95), however SD-OCT provided thicker values with a mean difference of 8.6 μm (Table 3, Fig 2A). Bland-Altman plots indicate that there were also fixed and proportional biases detected with differences in measurements between devices increasing with increasing mGCIPL thickness. A similar trend of fixed and proportional bias was found for comparing the sectoral mGCIPL thickness measurements from SS-OCT and SD-OCT, which are shown in Table 3.

**Table 3:** Agreement and correlation between mGCIPL, mGCC and cpRNFL measurements obtained using SS-OCT and SD-OCT.

	Mean difference (SD-OCT—SS-OCT) (μm)	95% CI of Agreement		P value^a^	Fixed Bias	Proportional Bias	P value^b^	R^2^
**mGCIPL**								
AVG	8.6	8.0	9.2	< 0.001	YES	YES	< 0.001	0.95
SUP	11.6	10.8	12.4	< 0.001	YES	YES	< 0.001	0.87
SN	9.8	9.3	10.3	< 0.001	YES	YES	< 0.001	0.94
ST	5.2	4.3	6.1	< 0.001	YES	YES	< 0.001	0.85
INF	11.1	10.1	12.0	< 0.001	YES	YES	< 0.001	0.83
IN	9.6	9.0	10.2	< 0.001	YES	YES	< 0.001	0.94
IT	4.5	3.5	5.5	< 0.001	YES	YES	< 0.001	0.82
**mGCC**								
AVG	6.5	6.0	7.1	< 0.001	YES	YES	0.003	0.95
SUP	9.7	9.0	10.5	< 0.001	YES	YES	< 0.001	0.94
SN	3.3	2.6	4.1	< 0.001	YES	NO	0.158	0.92
ST	7.1	6.5	7.7	< 0.001	YES	YES	< 0.001	0.94
INF	8.9	7.8	10.2	< 0.001	YES	YES	< 0.001	0.88
IN	4.1	3.1	5.1	< 0.001	YES	NO	0.604	0.88
IT	6.3	5.6	7.0	< 0.001	YES	YES	< 0.001	0.94
**cpRNFL**							
AVG	-1.0	-2.8	0.8	0.265	NO	YES	< 0.001	0.82
SUP	0.8	-1.5	3.1	0.516	NO	YES	< 0.001	0.78
INF	-0.2	-2.6	2.1	0.834	NO	YES	< 0.001	0.86
NAS	3.9	0.8	7.0	0.013	YES	YES	< 0.001	0.17
TEMP	-8.3	-9.9	-6.7	< 0.001	YES	YES	< 0.001	0.76

*a*. *P* values for mean difference

b. *P* values for the presence of correlation between the difference in pairwise comparison and their mean, after adjusting for inter-eye correlations.

Average mGCC thickness was 107.9 ± 8.1 μm versus 88.3 ± 12.8 μm in healthy and glaucomatous eyes respectively using SS-OCT (P<0.001) and 114.6 ± 7.2 μm versus 94.8 ± 14.7 μm respectively using SD-OCT (P<0.001) (Table 2). Similar to mGCIPL measurements, mGCC was thicker measured using SD-OCT compared to SS-OCT (Tables 2 and 3). For both SS-OCT and SD-OCT, subjects with glaucoma had significantly thinner mGCC than controls in all sectors (P < 0.001 for all sectors, Table 2). There was good correlation between mGCC thickness using SS-OCT and SD-OCT (R^2^ = 0.95), however, there was fixed bias in mGCC thickness between SS-OCT and SD-OCT in all average and sectoral mGCC measurements and proportional bias in all with the exception of superonasal and inferonasal sectors (Table 3, Fig 2B).

Average cpRNFL in healthy versus glaucomatous eyes was 102.4 ± 15.6 μm versus 71.1 ± 18.2 μm (P < 0.001) when measured by SS-OCT wide-angle scan, and 95.3 ± 8.6 versus 72.4 ± 12.1 μm (P < 0.001) when determined by SD-OCT optic disc cube scan. In healthy eyes, cpRNFL was thickest in the inferior sector and superior sectors and thinnest in the nasal and temporal sectors for both SS-OCT and SD-OCT (Table 2). Consistent with mGCIPL and mGCC readings, all the sectoral cpRNFL were significantly thinner in glaucomatous compared to healthy eyes using both devices (P <0.001 for all sectors). Fixed bias in cpRNFL measures was detected for nasal and temporal sectors, whereas proportional bias was present in all pairwise comparison between the two devices (Table 3, Fig 2C).

**Fig 3:**
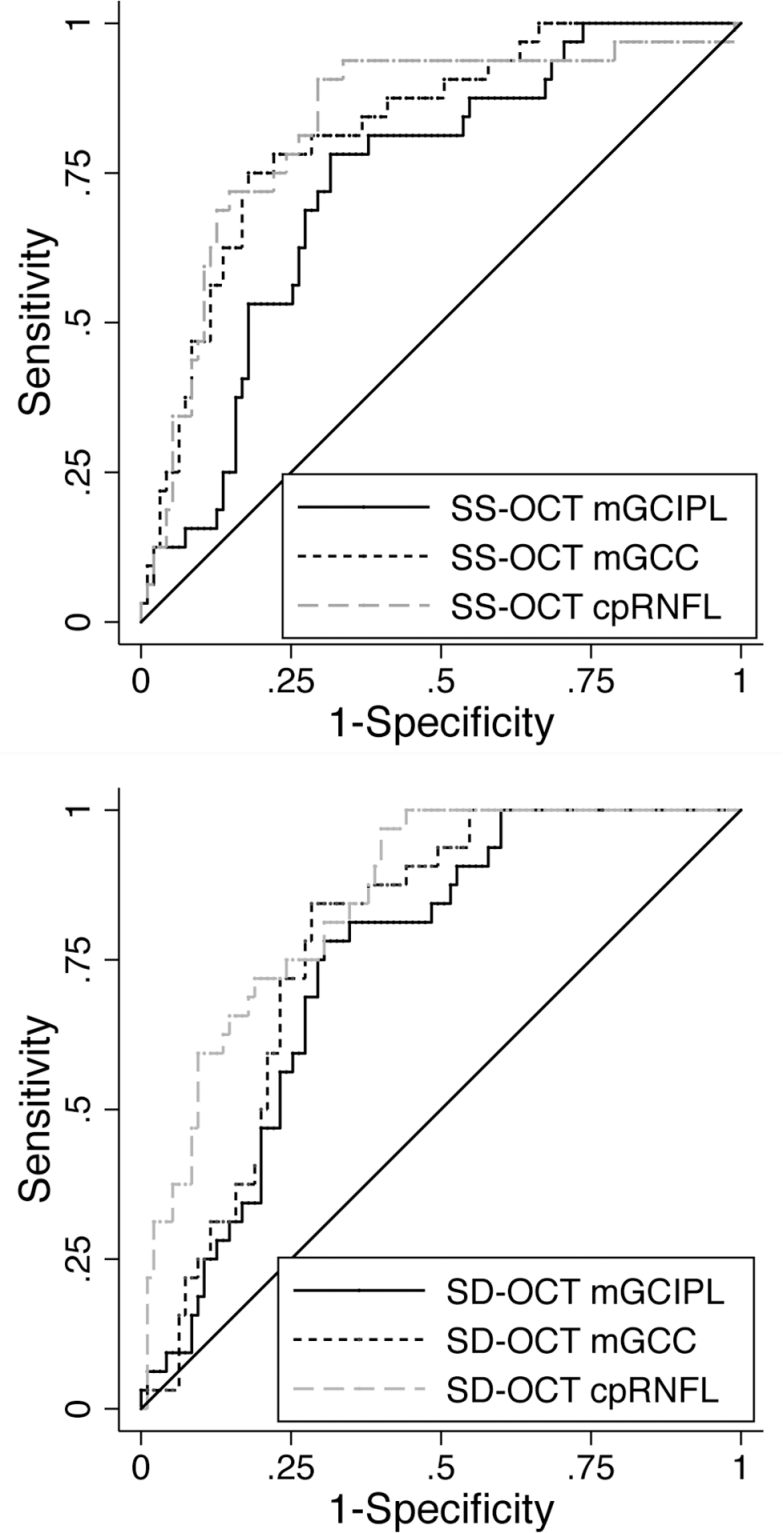
Receiver of operating characteristic curves showing the ability of mGCIPL, mGCC, and cpRNFL measured in swept source optical coherence tomography and spectral domain optical coherence tomography to distinguish healthy and glaucomatous eyes. Areas under the receiver operating characteristic curve (AUC) of mGCIPL, mGCC, and cpRNFL were 0.73, 0.82 and 0.83 by swept source optical coherence tomography (SS-OCT), respectively (top), and 0.75, 0.78, and 0.85 by spectral domain optical coherence tomography (SD-OCT), respectively (bottom).

AUCs showing the ability of SS-OCT and SD-OCT to distinguish eyes with and without glaucoma are presented in Table 4. Overall the best performing average parameters in both SS-OCT and SD-OCT were cpRNFL (AUC = 0.83 and 0.85 respectively, followed by mGCC (AUC = 0.78 and 0.82 respectively) and mGCIPL (AUC = 0.75 and 0.73 respectively. Minimum mGCIPL and mGCC measures also performed well (AUC = 0.86, Table 4), however it is only available in GCA analysis of SD-OCT. AUCs of average thickness of mGCIPL, mGCC and cpRNFL from SS-OCT and SD-OCT measurements are also shown in Fig 3.

**Table 4:** Age-adjusted areas under the receiver operating characteristic curves for differentiating healthy from glaucomatous eyes for mGCIPL, mGCC, and cpRNFL measurements obtained from SS-OCT and SD-OCT.

	Healthy vs. Glaucomamean (standard error)			Healthy vs. Early Glaucomamean (standard error)		
mGCIPL	SS-OCT	SD-OCT	*P* value	SS-OCT	SD-OCT	*P* value
AVG	0.73 (0.08)	0.75 (0.08)	0.561	0.67 (0.09)	0.67 (0.09)	0.817
SUP	0.69 (0.09)	0.71 (0.09)	0.665	0.66 (0.11)	0.64 (0.10)	0.624
SN	0.66 (0.09)	0.63 (0.09)	0.280	0.63 (0.11)	0.61 (0.11)	0.489
ST	0.73 (0.08)	0.77 (0.08)	0.113	0.64 (0.09)	0.69 (0.09)	0.247
INF	0.71 (0.08)	0.74 (0.08)	0.494	0.66 (0.09)	0.68 (0.10)	0.714
IN	0.65 (0.08)	0.63 (0.09)	0.340	0.61 (0.09)	0.59 (0.10)	0.276
IT	0.81 (0.06)	0.83 (0.05)	0.500	0.77 (0.07)	0.78 (0.07)	0.765
MIN	N/A	0.86 (0.06)	N/A	N/A	0.82 (0.07)	N/A
**mGCC**						
AVG	0.82 (0.07)	0.78 (0.07)	0.165	0.75 (0.08)	0.71 (0.09)	0.227
SUP	0.77 (0.08)	0.72 (0.09)	0.087	0.70 (0.09)	0.65 (0.10)	0.260
SN	0.71 (0.10)	0.67 (0.10)	0.148	0.65 (0.11)	0.64 (0.11)	0.725
ST	0.82 (0.07)	0.79 (0.08)	0.215	0.73 (0.08)	0.70 (0.09)	0.440
INF	0.80 (0.07)	0.77 (0.07)	0.480	0.73 (0.08)	0.72 (0.09)	0.813
IN	0.75 (0.09)	0.69 (0.08)	0.124	0.67 (0.10)	0.63 (0.10)	0.341
IT	0.84 (0.05)	0.82 (0.06)	0.382	0.78 (0.06)	0.75 (0.07)	0.369
MIN	N/A	0.86 (0.06)	N/A	N/A	0.81 (0.08)	N/A
**cpRNFL**						
AVG	0.83 (0.06)	0.85 (0.05)	0.609	0.81 (0.07)	0.81 (0.06)	0.925
SUP	0.87 (0.06)	0.90 (0.04)	0.377	0.85 (0.06)	0.88 (0.05)	0.374
INF	0.79 (0.07)	0.82 (0.07)	0.487	0.75 (0.08)	0.76 (0.09)	0.641
NAS	0.59 (0.08)	0.55 (0.08)	0.681	0.60 (0.08)	0.50 (0.09)	0.295
TEMP	0.69 (0.08)	0.65 (0.09)	0.357	0.63 (0.10)	0.62 (0.10)	0.794

Among sectoral mGCIPL measurements, the sector showing highest diagnostic ability for glaucomatous damage was the inferotemporal sector (AUC = 0.81 for SS-OCT and 0.83 for SD-OCT). The worse performing mGCIPL sector was the inferonasal sector for SS-OCT (AUC = 0.65), and inferonasal and superonasal sectors for SD-OCT (AUC = 0.63). The inferotemporal sector was also the best performing mGCC sector (AUC = 0.84 for SS-OCT and 0.82 for SD-OCT) and the worse performing mGCC sector was the superonasal sector (AUC = 0.71 vs. 0.67 for SS-OCT and SD-OCT respectively). The best performing cpRNFL sector was the superior sector (AUC = 0.87 for SS-OCT, and. 0.90 for SD-OCT) and the worse performing the nasal sector (AUC = 0.59 r SS-OCT and. 0.55 for SD-OCT). There was no significant difference between SS-OCT and SD-OCT in AUCs of average and sectoral mGCIPL, mGCC and cpRNFL measurements (Table 4).

As diagnostic ability depends on the stage of disease, we also examined the ability of the macula and circumpapillary measurements to distinguish healthy eyes from those with early glaucoma (defined as SAP MD ≥ -6dB, Table 4). The best performing parameters for early disease was minimum mGCIPL measured with SD-OCT (AUC = 0.82), and superior cpRNFL (AUC = 0.85 with SS-OCT and 0.88 with SD-OCT). Average cpRNFL also performed well on both devices (AUC = 0.81).

There was no significant difference in AUCs when mGCIPL and mGCC were measured using SD-OCT, however with SS-OCT mGCC measurements tended to have small but significantly better glaucoma discrimination ability than mGCIPL for average, superior, superotemporal, and inferior sectors (P < 0.05, Table 5). Compared to average cpRNFL, no difference in glaucoma diagnostic ability was found for either average mGCIPL or mGCC (Table 5). However, for detection of early glaucoma, cpRNFL measurement had significantly better diagnostic ability than mGCIPL using SS-OCT but not SD-OCT (Table 5).

**Table 5:** P-values comparing areas under the receiver operating characteristic curves for the ability of mGCIPL versus mGCC to differentiate healthy and glaucomatous eyes.

	Glaucoma		Early Glaucoma	
	P value (SS-OCT)	P value (SD-OCT)	P value (SS-OCT)	P value (SD-OCT)
	**mGCIPL vs. mGCC**			
**AVG**	0.005	0.102	0.030	0.247
**SUP**	0.033	0.758	0.246	0.661
**SN**	0.219	0.353	0.697	0.520
**ST**	0.009	0.397	0.073	0.591
**INF**	0.035	0.170	0.090	0.185
**IN**	0.069	0.086	0.290	0.172
**IT**	0.340	0.396	0.662	0.332
**MIN**	N/A	0.943	N/A	0.788
**mGCIPL vs. cpRNFL**				
**AVG**	0.061	0.086	0.026	0.103
**mGCC vs. cpRNFL**				
**AVG**	0.867	0.238	0.324	0.202

P-values comparing areas under the receiver operating characteristic curves for mGCIPL versus cpRNFL, and mGCC versus cpRNFL are also shown. All thickness measurements were obtained using both SS-OCT and SD-OCT.

Abbreviations: SS-OCT = swept source optic coherence tomography; SD-OCT = spectral domain optic coherence tomography; mGCIPL = macular ganglion cell inner plexiform layer; mGCC = macular ganglion cell complex; cpRNFL = circumpapillary retinal nerve fiber layer; AVG = average; SUP = superior; INF = inferior; NAS = nasal; TEMP = temporal; SN = superonasal; IN = inferonasal; ST = superotemporal; IT = inferotemporal; MIN = minimum.

## Discussion

In the present study, we evaluated the ability of two related macular measures, mGCIPL and mGCC, to differentiate glaucomatous from healthy eyes using two image acquisition modalities, SS-OCT wide-angle scan and SD-OCT macular scan and compared the results to cpRNFL thickness. Our results showed that the thickness of both mGCIPL and mGCC was significantly reduced in glaucomatous compared to healthy eyes, with both parameters showing similar ability to detect glaucoma as cpRNFL. Furthermore, the good diagnostic performance of cpRNFL, mGCIPL and mGCC was maintained for early glaucoma (MD ≥ -6dB). These findings indicate that both mGCIPL and mGCC are potentially useful measures for the identification of glaucomatous damage, even at early stages of disease. Individual measures with the highest glaucoma discrimination ability were SD-OCT measured minimum mGCC (AUC = 0.86), minimum mGCIPL (AUC = 0.86), and superior cpRNFL (AUC = 0.90).

Although average and sectoral mGCIPL thicknesses were measured with two different scan protocols of two different OCT devices, the ability to detect glaucoma was similar. Among all average and sectoral mGCIPL measures (except minimum thickness in SD-OCT), the range of AUCs using both OCT devices (0.65–0.81 for SS-OCT, and 0.63–0.83 for SD-OCT, respectively) were remarkably similar. However, between the two OCT devices we were able to detect both fixed and proportional bias for each corresponding mGCIPL measure, which reflect systemic differences between the two imaging modalities, with the result that readings from SS-OCT and SD-OCT are not interchangeable.

The macular parameter with the best overall performance for distinguishing healthy and glaucomatous eyes were SD-OCT minimum mGCIPL and mGCC thickness, which achieved an AUC of 0.86 and were also good at detecting eyes with early glaucoma (AUC = 0.82 for mGCIPL, and 0.81 for mGCC). This observation is consistent with previous reports in which minimum mGCIPL and minimum mGCC thickness were among the best performing OCT parameters for glaucoma discrimination.[31,32] While significant glaucomatous retinal ganglion cell loss is detectable by measuring average mGCIPL or mGCC thickness, which are a cross-sections of the inner retina that contain only a few layers of retinal ganglion cells and their associated synaptic and axonal structure, focal changes in mGCIPL or mGCC thickness in early glaucoma may be masked by averaging measurements from a large area, for example, through calculation of global or sectoral thicknesses. Minimum mGCIPL or mGCC thickness may allow better identification of focal neural losses and therefore earlier detection of disease.

Initial studies examining the role of macular thickness measurements in glaucoma found that quantification of full retinal thickness was helpful for glaucoma diagnosis.[7,33–38] However, as thickness of the outer retina is largely unchanged in glaucoma, inclusion of outer retinal layers, such as the outer plexiform layer, outer nuclear layer and photoreceptor segment layers, may reduce the sensitivity with which glaucomatous damage can be detected. Several investigators have shown mGCC, which includes mGCIPL and RNFL, to have improved diagnostic performance compared to full macular thickness, with an AUC similar to that of cpRNFL thickness. [15,17,39,40]

To determine whether mGCIPL may be useful in glaucoma diagnosis, Wang et al. manually segmented mGCC to isolate mGCIPL, and demonstrated that mGCIPL thickness were thinner in eyes of glaucoma patients.[41] Automated algorithms for mGCIPL segmentation are now available and were shown to be associated with good glaucoma discrimination ability. The present study confirmed these results;[32] we found, although mGCIPL had good diagnostic accuracy, no significant improvement in diagnostic performance of mGCC compared to mGCIPL using either SS-OCT or SD-OCT.

In agreement with previous studies, we found mGCIPL and mGCC had largely similar glaucoma diagnostic ability to cpRNFL.[13–17] Despite this, macular measurements may offer a few advantages in glaucoma diagnosis compared to cpRNFL. First, macular measurements have been reported to be less variable than cpRNFL.[42–44] As a flat structure at posterior pole, the macula is less subject to interference from central retinal vessels, peripapillary atrophy, and precision in circle placement around the disc. A further possible advantage of macular measurements is that they, along with related central visual field testing, may offer a broader dynamic range of measurement to follow progression in advanced glaucoma.[45] However, it is important to note that macular co-morbidities, such as diabetic retinopathy, retinal vascular occlusive diseases, epiretinal membrane and vitreomacular traction can also alter macular thickness. Although we excluded eyes with these conditions in the present study, these conditions may limit the usefulness of mGCC and mGCIPL in some patients.

Measures of cpRNFL and macular GCC/GCIPL can be acquired with two separate scans, or in a single wide-field scan. Acquiring macular and optic nerve head measurements from a single scan is potentially less time consuming, and minimizes problems with alignment if comparing macular and circumpapillary data.

In the present study, we evaluated whether macular measures obtained from a wide-angle scan can offer similar diagnostic performance as measures obtained from a separate macula cube scan. Our results showed that average and sectoral macular and cpRNFL measures acquired from SS-OCT wide-angle scan performed similarly well as corresponding measures obtained from separate macular and disc cube scans by SD-OCT. On the other hand, we must keep in mind the potential differences between images acquired with wide-angle scan protocol versus disc circle scan or macula scan. According to our data, cpRNFL thickness measured by SS-OCT wide-angle scan appear to be thicker than those measured by SD-OCT, whereas mGCIPL measurements are smaller comparing to the corresponding measures by SD-OCT. (Table 2). Besides the differences in the segmentation algorithms utilized by the two OCT devices, other factors such as scan quality may also contribute to the small discrepancies in cpRNFL and mGCIPL measurements. In wide-angle scan images of posterior pole are taken by focusing on the region between the optic disc and macula.[23] This approach may cause the laser angle slightly different from the one used in conventional optic disc or macula scan, which is centered on the disc or fovea. Since the nerve fiber layer reflectance can be highly affected by the angle of illumination, difference in focusing areas during image acquisition between wide-angle scan in SS-OCT and disc or macula scans using SD-OCT may have an impact on their cpRNFL readings, although it remains to be determined whether macular measures such as mGCIPL and mGCC thicknesses are affected as well.[46] Another limitation of wide-angle scan may be associated with sparser sampling over 12 X 9 mm area. However, the improved scan speed with SS-OCT allows a sampling density of 1213.6 (512 X 256 /12 X 9) axial scans per mm^2^ in its wide-angle scan protocol, whereas macula cube scan with Cirrus HD-OCT samples 1111.1 (200 X 200 / 6 X 6) axial scans per mm^2^. Despite these limitations of wide-angle scanning, the current results suggest that wide-angle scan can offers a similar diagnostic value while reducing the time that patients have to spend on adjunctive imaging in the glaucoma clinic.

The present study has limitations. A limitation of this study does not evaluate the diagnostic performance of mGCIPL and mGCC measures in the context that closely resembles the clinical setting. Other co-morbidities such as diabetic retinopathy and retinal vascular occlusive diseases can also cause significant changes in the thickness of macular layers, and they often involve those patients of the similar age group as glaucoma. With the goal of evaluating the ability of differentiating healthy from glaucomatous eyes, we excluded eyes with co-existing pathologies that affect macular thickness. This approach will certainly limit our ability to directly generalize the glaucoma diagnostic accuracy using mGCIPL and mGCC measurements established in this study into actual glaucoma clinic populations. Secondly, this cross-sectional study did not evaluate how well the different devices, and scan patterns can identify glaucomatous progression; this remains to be determined with longitudinal study. Additionally, the correlation between the structural measurements with SS-OCT and SD-OCT was determined by Pearson correlation with inter-eye correction. However, similar inter-eye correction cannot be performed in Bland-Altman agreement analysis. Lastly, there was a significant difference in age distributions between healthy controls and glaucoma patients in this cohort. For this reason we adjusted for age in the ROC analysis. However, Bland-Altman analysis cannot be adjusted for age, and this should be taken into consideration when interpreting the results.

In conclusion, our analysis demonstrated that, similar to cpRNFL, both mGCIPL and mGCC offer good ability to differentiate healthy and glaucomatous eyes, with SS-OCT wide-angle scan providing mGCIPL and mGCC measures as useful as SD-OCT macular scan. In addition we found since there are differences in the thickness values measurements of mGCIPL and mGCC obtained with SD-OCT and SS-OCT, the measurements should not be used interchangeably in patient management decisions.

## Supporting Information

S1 Table Dataset of SS-OCT versus SD-OCT. The dataset used in this study contains mGCIPL, mGCC and cpRNFL measures by SS-OCT and SD-OCT from 106 eyes of glaucoma patients and 41 eyes of healthy participants.
(CSV)
